# Himalayan dock (*Rumex nepalensis*): the flip side of obnoxious weed

**DOI:** 10.1186/s40781-015-0067-z

**Published:** 2015-11-05

**Authors:** Kesang Wangchuk

**Affiliations:** Renewable Natural Resources Research and Development Center, Bumthang, 32001 Bhutan

**Keywords:** Acid detergent fiber, Anti-nutrient, Crude protein, Dry matter, Neutral detergent fiber, *Rumex nepalensis*

## Abstract

Himalayan dock (*Rumex nepalensis*) was evaluated for forage value and antinutrients under three, five and seven weeks cutting intervals in the temperate environment. Dry matter (DM) content was measured for each cutting interval. Forage quality parameters such as Crude Protein (CP), Acid Detergent fiber (ADF), Neutral Detergent Fiber (NDF), Calcium (Ca) and Phosphorus (P) were analyzed. Plants with seven weeks cutting interval gave higher DM yield. CP and P content were significantly higher for three weeks cutting intervals. Average CP contents were 31.38 %, 30.73 % and 27.32 % and average P content 0.58 %, 0.52 % and 0.51 % for three, five and seven weeks cutting intervals, respectively. Ca content did not differ significantly between cutting intervals. The average Ca content were 0.91 %, 0.90 % and 90 %, for three, five and seven weeks cutting intervals, respectively. Tannin and mimosine contents were not significantly different between cutting intervals. Average tannin contents were 1.32 %, 1.27 % and 1.26 % and mimosine 0.38 %, 0.30 % and 0.28 % for three, five and seven weeks cutting intervals, respectively. The study concluded that *R. nepalensis* could be a potential source of protein for livestock. The study also suggests seven weeks harvesting interval to provide plants with high dry matter yield, high forage quality and very low levels of anti-nutrients.

## Background

*Rumex nepalensis* or the Himalayan dock belongs to the family Polygonaceae. It is commonly sighted at higher altitudes and grows between 900–4000 m on moist as well as dry slopes, under shades, and even in plains. *R. nepalensis* have broad ecological tolerances. It is a common weed in pastures and the plants are known to become dominant and outcompete desirable pasture species and degrade pasture quality. It regenerates from tap roots and establishes quickly as seedlings. Once established, tough tap roots become difficult to remove and are not readily killed by tillage. These characteristics have often made farmers to regard this species as the most difficult weed [1]. The general issues of *Rumex spp* are that they are weeds of grassland, particularly on disturbed areas with high fertility.

However, available literatures suggest that this species possess other characteristics. From the forage perspective, Humaira et al. [2] noted the moderate palatability of leaves of *R. nepalensis* to goat. At the young phenological stages of growth, the plants of *R. nepalensis* are leafy and Al Haj Khaled et al. [3] positively associated leafiness to higher crude protein (CP) and digestibility. While the leaf CP of matured *R. nepalenses* is about 14 % [4], the leaf CP of young plants of this species is over 32 % [5]. These reports highlight difference in CP content of this species according to phenological growth stages besides indicating that the young plants of *R. nepalensis* could be a good source of protein. Despite the higher CP content, *R. nepalensis* has been least studied for its forage value. Further, there is also a lack of information on the anti-nutritive properties of *R. nepalensis*. Anti-nutritional factors in animal feed are known to reduce the availability of one or more nutrients to the animal.

Feed represents the largest single production expense for cattle production. Protein is a critical dietary nutrient in all cattle feed, yet the high cost of commercial protein supplements is one of the main limitations to efficient animal production by smallholders [6]. On the contrary, the pasture based cattle production is relatively cheaper, however, the cattle farmers must ensure that the forages are supplemented with adequate protein and energy to meet the nutritional demands of animals. In such scenario, the use of protein rich plant sources can reduce demand for protein ingredients from expensive commercial feed sources. Thus, protein from plant resources can reduce the overall production cost. To generate estimates of forage quality and anti-nutritive factors, a study was conducted with the objectives to evaluate difference in forage value and anti-nutritive properties between three phenological growth stages of *R. nepalensis* in the Himalayan highland.

## Methods

### Experimental site

The study was conducted in Bumthang valley in central Bhutan. Bumthang lies at an altitude of 2700 m and is one of the focal districts for pasture development in Bhutan. The valley is situated in the north central temperate region with an area of about 2715 km^2^ [7]. Most pasture fields are on rugged terrain and normally spread across slopes with gradients ranging from 0–35° [8]. The climate is dominated by the Indian monsoon with high levels of precipitation between June and September followed by cold and dry winter. The mean maximum precipitation of 152 mm is recorded in the month of July and August, and the mean maximum temperature of 22 °C is recorded in June and the mean minimum temperature of −2.7 °C in January [8]. The vegetation growing period commences from May and peaks in August with gradual decline in temperature from September. Winter is cold and dry from November to March.

### Description of species under study

*R. nepalensis* is a perennial herb. Roots are large and stems erect. The plants are 50–100 cm tall. Basal leaves have petiole length of 4–10 cm. Leaf blade is broadly ovate. Inflorescence is paniculate and flowers are bisexual. The plants grow in the altitude range of 900–4000 m and are commonly seen on grassy slopes, moist valleys and along ditches.

### Experimental design and treatments

The experimental design was a randomized complete block. The treatment was cutting interval of three types, three, five and seven weeks intervals with five replications. The experiment was established by demarcating plots on existing vegetation of *R. nepalensis*. The individual plot size was 1 m^2^. Spacing were 50 cm between plots and 70 cm between replications. The plants were grown under natural conditions without fertilizer application. The treatments were applied on June 25, July 8 and July 22 for three, five and seven weeks cutting intervals, respectively.

### Estimation of DM, forage value and anti-nutrients

Plants in each plot were clipped and weighed for fresh weight. The clipped samples were processed and oven dried at 60 °C for 48 h. Dried sample weight was recorded and the DM per hectare area was estimated. Following DM estimation, the dried samples were processed and analyzed for total nitrogen (N). The total nitrogen content in the sample was determined with Kjeldahl method and CP was estimated as % N × 6.25. The estimate was compared with the CP content of other high quality forages. ADF was estimated following the procedures described by Goering and Van Soest [9] and NDF with methods of Van Soest [10]. Ca and P were estimated with methods of AOAC [11].

The anti-nutrients estimated were mimosine and tannin. Mimosine content was estimated with the calorimetric method described by Matsumoto and Sherman [12] while tannin content was estimated using vanillin hydrochloric acid method of Burn [13].

### Data analysis

The dataset was tested for normalcy and homogeneity of variances using Shapiro-Wilk and Levene’s tests, respectively. Wherever required, data was logarithmically transformed to meet the assumptions of ANOVA. Differences in the means of morphological traits, CP, Ca, P and anti-nutrients between cutting intervals were assessed with several one-way ANOVAs. Differences between means were considered significant if *P* values were lower than 0.05. The entire dataset was analysed with SPSS 22 [14].

## Results

### Morphological traits, Crude protein and anti-nutrients

**Fig. 1:**
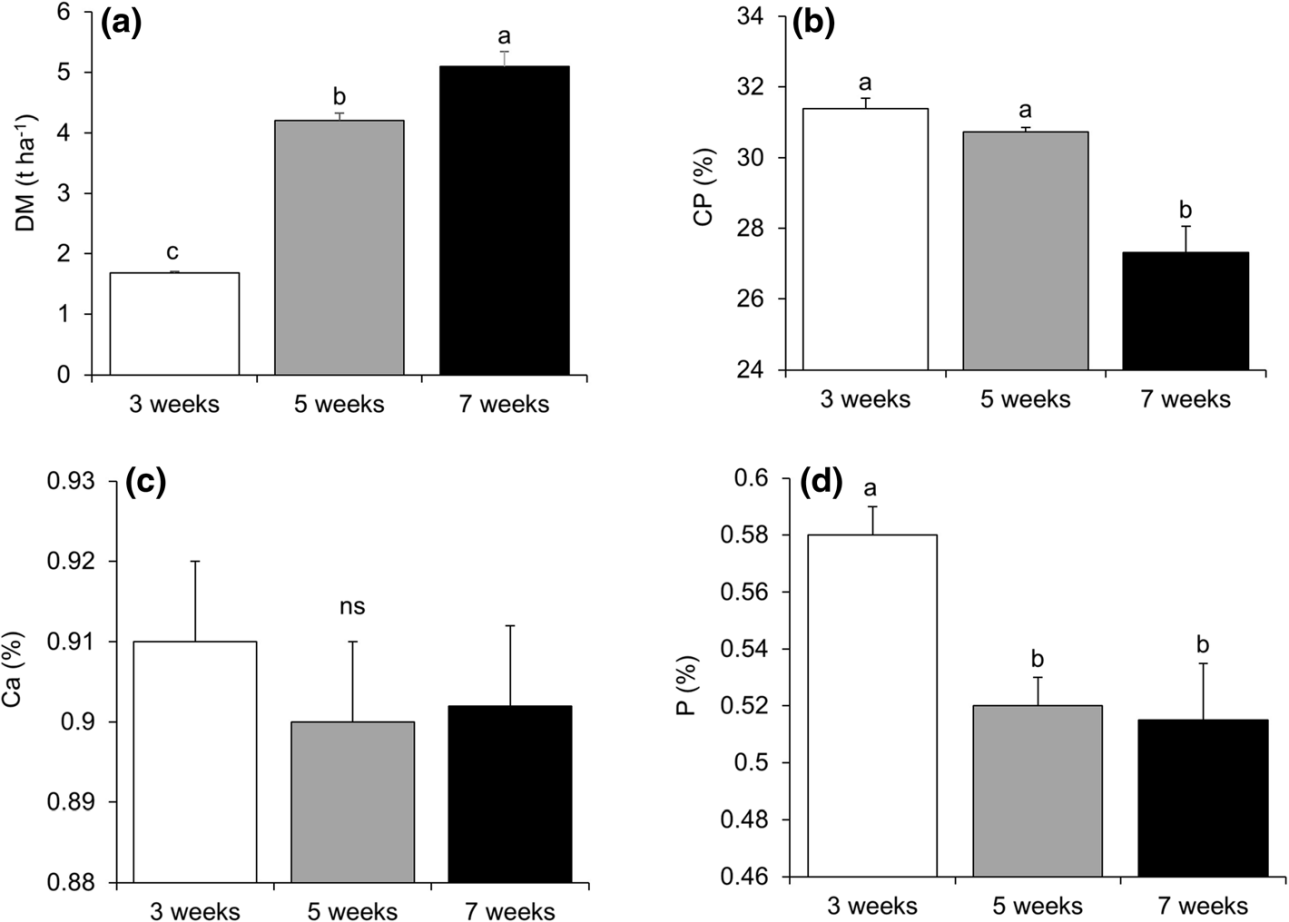
**a** Dry matter (DM) **b** Crude protein (CP) **c** Calcium (ca) and **d** Phosphorus (P) of *R. nepalensis* under 3, 5 and 7 weeks cutting intervals

DM content was significantly higher for plants receiving 7 week cutting interval (Fig. 1a). CP in actively growing plants of *R. nepalensis* varied among cutting intervals. CP declined with increasing cutting interval. CP content was significantly lower for the plants subjected to seven weeks cutting interval than those under three and five weeks cutting intervals (Fig. 1b). Ca level was not significantly different between cutting intervals (Fig. 1c). However, P was significantly greater for *R. nepalensis* under three weeks cutting intervals (Fig. 1d).

**Fig. 2:**
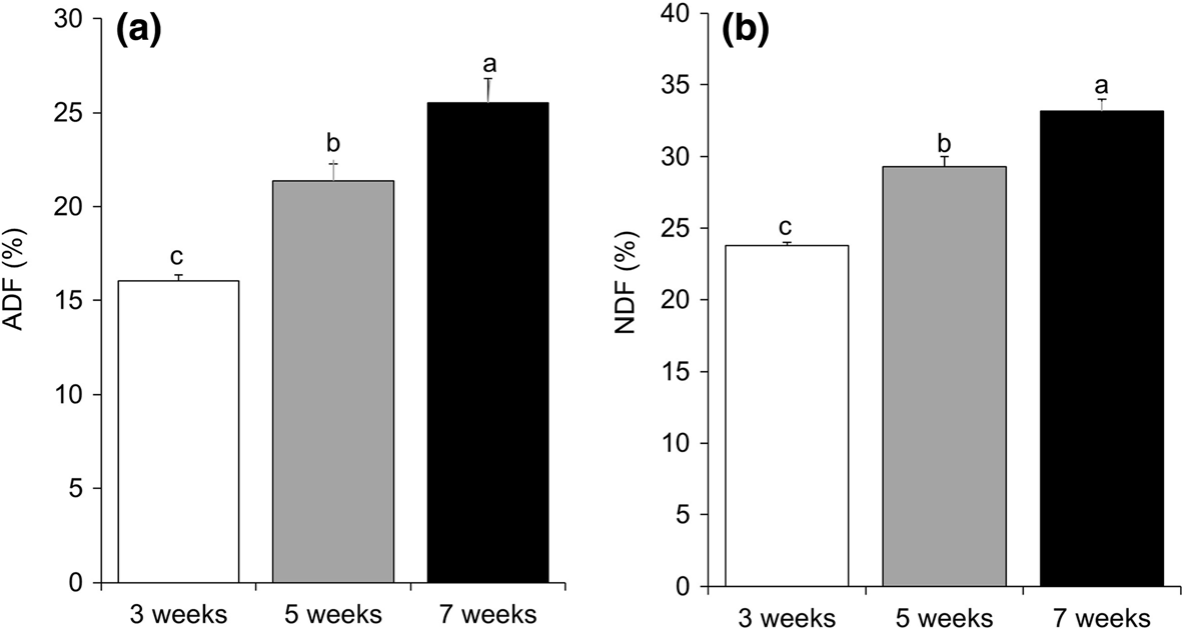
**a** ADF and **b** NDF of *R. nepalensis* under 3, 5 and 7 weeks cutting intervals

**Fig. 3:**
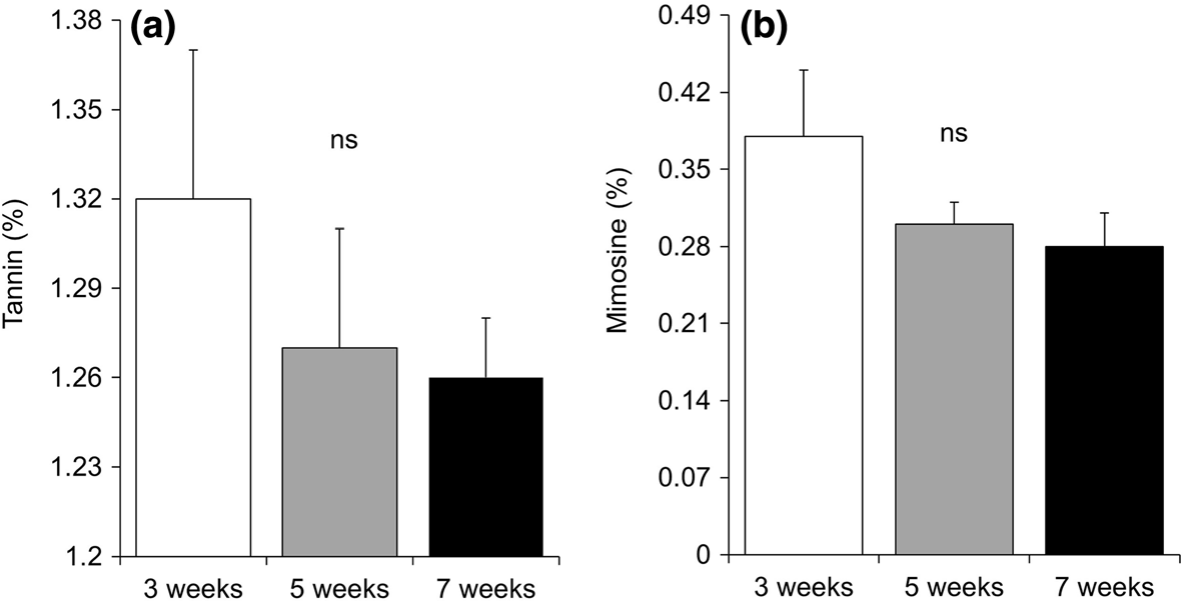
**a** Tannin and **b** Mimosine of *R. nepalensis* under 3, 5 and 7 weeks cutting intervals

Both ADF and NDF were significantly higher for plants under seven weeks cutting interval (Fig. 2a and b). Although non-significant, the tannin and mimosine content were higher for the plants subjected to three weeks cutting interval (Fig. 3a and b).

Comparison with other important feed resources revealed *R. nepalensis* to possess greater level of CP, Ca and P than white clover and willow leaves (Table 1). However, the CP, Ca and P contents were comparable with the Karma feed, a commercial feed concentrate known to be of higher quality.

**Table 1:** Comparison of CP content of different feed and forage resources

Feed resource	CP %	Ca %	P %	Ca:P
1. Karma concentrate feed (starter)	21.69^a^	0.86^a^	0.58^a^	1.4:1
2. Karma concentrate feed (Finisher)	20.59^a^	0.75^a^	0.47^a^	1.6:1
3. White clover	18.00^b^	1.75^d^	0.33^d,c^	5.3:1
4. Willow leaves	16.00^b^	2.02^e^	0.28^e^	7.2:1
5. *R. nepalensis* (3 weeks old)	31.38^c^	0.91^c^	0.58^c^	1.6:1
6. *R. nepalensis* (5 weeks old)	30.73^c^	0.90^c^	0.52^c^	1.7:1
7. *R. nepalensis* (7 weeks old)	27.32^c^	0.90^c^	0.52^c^	1.7:1

^a^Wangchuk and Nidup [19], ^b^Roder *et al.* [26], ^c^This study, ^d^Frame and Newbould [27], ^e^Upreti and Shrestha [15]

## Discussion

### CP, fiber and mineral content

The present study shows that cutting intervals below five weeks provide CP over 30 %, which is closer to the leaf CP content of 32 % reported by Abbasi et al. [5]. Although high in CP, the short intervals are disadvantaged by low DM yield. Amongst cutting intervals, the seven weeks interval appears to provide optimum CP and higher DM yield. From forage standpoint, seven weeks seem to be an optimal cutting interval for *R. nepalensis*, from which a balance between forage yield and quality can be expected. CP content of seven weeks interval is about 27 %, which translates to 270 g CP kg^−1^ DM. Upreti and Shrestha [15] categorize feeds and forages with CP 20 % and above as high quality roughages and *R. nepalensis* falls in this category. This evidently highlights the potential of *R. nepalensis* to make valuable contributions to the nutritive value of forage-based diets. According to Topps [16], if forage legume is used as protein source for milk production in grass based diet the legume should contain not less than 160 g CP kg^−1^ DM to obtain a desirable protein level of 100 g CP per kg DM in the final diet. Ruminants require minimum of 150 g CP kg^−1^ DM for lactation and growth [17]. For the early lactating cows, Linn [18] recommends ration containing 18-19 % CP or 180–190 g CP kg^−1^ DM. The CP of *R. nepalensis* is much higher than the required level and in excess in the range of 80–110 g CP kg^−1^ DM, which strongly suggests that *R. nepalensis* can be a protein rich source to support milk production. However, harvest beyond seven weeks interval may cause further decline in CP content below 20 %, which will categorize plants as moderate quality roughages [15].

The CP percentage of *R. nepalensis* is higher than the other important temperate forage resources such as white clover, willow leaves and grass-clover pasture, but compares well with Lucerne hay and Karma feed, the popular concentrate feed in Bhutan. This further reiterates that *R. nepalensis* if harvested at early stages of growth could be a protein rich supplement in cattle feed. Thus, *R. nepalensis* has potential to provide alternative low-cost protein to meet the nutritive requirements of cattle in smallholder farms. However, owing to low DM yield, it is likely that the CP yield in terms of kg ha^−1^, might be lower than other feed resources. This could be a disadvantage when the demand for dietary protein is high. Martens et al. [6] asserts that, in the smallholder farms, fast growth rates demand an optimum supply of dietary protein, which may not be met by the locally-grown feeds.

Both ADF and NDF content increased with increasing cutting intervals with highest content under seven weeks cutting interval. However, the values were below 31 % and 40 % for ADF and NDF, respectively. According to Upreti and Shrestha [15], forages with ADF below 31 % and NDF below 40 % are categorized as roughages with good digestibility. This highlights that *R. nepalensis* can still have good digestibility even when harvested at seven weeks interval. However, similar to CP, the cutting interval greater than seven weeks may be less desirable as it is likely to cause decline in digestibility below the desired level.

The calcium and phosphorus content under seven weeks cutting interval were about 0.90 % (9 g kg^−1^ DM), 0.52 % (5.2 g kg^−1^ DM), respectively. For grasses and forage legumes, Upreti and Shrestha [19] categorize Ca content below 2 % as low and P content above 0.5 % as high. Therefore, the Ca content of *R.nepalensis* must be considered low and inadequate to be included in the ration of a lactating cow that requires a minimum of 0.75 % Ca whereas P level is high enough [18].

### Anti-nutrients

Tannin and mimosine were two main antinutrients present in *R. nepalensis*. Tannins has been found to reduce the digestibility of protein and carbohydrates including starch and fibers, and has bitter and astringent taste, which in many cases reduces palatability and can depress growth [6]. In this study, the tannins in *R. nepalensis* under all cutting intervals were below 1.3 %, which translates to 13 g kg^−1^ DM. The amount is far below than the total tannin content of oak leaves (*Quercus semecarpifolia*) (78 g kg^−1^ DM) [20], an important tree fodder in the Himalayan region. According to Barry and Manley [21], forage containing more than 5 % tannins are tannins rich forages. Tannins with 5 to 10 % in the feed are antinutritive and toxic [22]. The tannin level of *R. nepalensis* must be considered very low. In contrast to the toxic effects of high tannin level, the low tannin level has been reported to have beneficial effects. Low tannin level protects protein of forages and allows a high efficiency of feed utilization by the animal [23]. This likely explains why there has been no field reports of animal poisoning by this species.

Mimosine is the second antinutrient found in *R. nepalensis*. Under all cutting intervals, the mimosine level was below 0.38 %, which translates roughly to 3.8 g kg^−1^ DM. The toxic effects are found to manifest when levels of mimosine in diets exceed 0.015 % of animal’s body weight [24]. In terms of DM weight, the toxic amount calculates to 45 g per 300 kg body weight, which is the standard live body weight of one livestock unit consuming 6 kg dry matter in the Bhutan Himalaya [25]. If one livestock unit feeds on *R. nepalensis* vegetation, the amount of mimosine likely to be consumed estimates to just over 20 g per 6 kg DM. This is far below the lethal amount and very unlikely to cause toxic effects.

## Conclusion

*R. nepalensis* has high CP content under all cutting intervals but seven weeks interval provides high dry matter yield. High CP, low fiber and P content except Ca qualify *R. nepalensis* to be graded as a prime feed and a potential source of protein. Level of antinutrients is very low and plants are safe for use as protein supplement. The results of this study have value for farmers and development workers. Although high in overall quality, there is a need for further studies on the effects of feeding *R. nepalensis* on milk production. Further, the combination of this species with other feeds should also be studied to establish optimum levels of inclusion to maximize feed intake. Finally, rather than viewing *R. nepalensis* as difficult weed, much would be gained if it is utilized as a protein supplement for livestock.
